# Bringing Culture to the Uncultured: *Coxiella burnetii* and Lessons for Obligate Intracellular Bacterial Pathogens

**DOI:** 10.1371/journal.ppat.1003540

**Published:** 2013-09-05

**Authors:** Anders Omsland, Ted Hackstadt, Robert A. Heinzen

**Affiliations:** 1 Host-Parasite Interactions Section, Laboratory of Intracellular Parasites, Rocky Mountain Laboratories, National Institute of Allergy and Infectious Diseases, National Institutes of Health, Hamilton, Montana, United States of America; 2 Coxiella Pathogenesis Section, Laboratory of Intracellular Parasites, Rocky Mountain Laboratories, National Institute of Allergy and Infectious Diseases, National Institutes of Health, Hamilton, Montana, United States of America; University of North Carolina at Chapel Hill School of Medicine, United States of America

## Introduction

Human diseases caused by obligate intracellular bacterial pathogens result in significant morbidity and mortality. Obligate intracellular bacteria that replicate in the cytoplasm of host endothelial cells include *Rickettsia prowazekii, Rickettsia rickettsii*, and *Orientia tsutsugamushi* (etiologic agents of epidemic typhus, Rocky Mountain spotted fever, and scrub typhus, respectively). Residing in specialized vacuolar compartments are *Anaplasma phagocyophilum* and *Ehrlichia chaffensis* (agents of febrile illnesses that have tropisms for neutrophils and monocytes, respectively) and *Chlamydia trachomatis*, which targets mucosal epithelia and causes blinding trachoma and sexually transmitted diseases. *Coxiella burnetii* preferentially colonizes mononuclear phagocytes during natural infection where it inhabits a specialized vacuole with properties of a phagolysosome [1]. The pathogen causes a debilitating influenza-like illness in humans called Q (query) fever, a disease that has received recent notoriety due to a large outbreak in the Netherlands [2]. The absolute reliance of obligates on a eucaryotic host cell for growth imposes significant experimental constraints, not the least of which is difficulty in establishing pathogen genetic systems. However, *C. burnetii* was recently liberated from its host cell by a medium that supports axenic (host cell–free) growth. Here, we provide a brief overview of the systematic approach used in *C. burnetii* media development and discuss how insight gained from this success could facilitate development of axenic media for other obligate intracellular bacterial pathogens.

## Known Physiology and Cellular Microbiology Greased the Wheels for *C. burnetii* Axenic Media Development

Prior metabolic studies of host cell–free *C. burnetii* and knowledge of pathogen-host interactions provided a foundation on which to base initial media formulations. The critical finding that the *Coxiella*-containing vacuole (CCV) resembles a phagolysosome [3] led to several reports showing metabolic activity of purified bacteria was optimal under moderately acidic conditions (approx. pH 5) [4]. The mechanistic basis of “acid activation” of *C. burnetii* metabolism is unresolved although it may involve stimulation of proton symporters [5]. Pathogen-host interactions revealed that the CCV is highly fusogenic with fluid phase endosomes, but impermeable to small molecules within the cytosol [1], suggesting the ion composition of the CCV might reflect that of the serum/tissue culture medium, i.e., low concentration of K^+^ (∼5 mM) and high concentrations of Na^+^ (∼145 mM) and Cl^−^ (∼110 mM). Furthermore, the CCV fuses with autophagosomes, a process predicted to deliver proteinacious material that can be degraded into peptides and amino acids by the hydrolytic activity of the vacuole [6]. Indeed, early acid activation studies showed a preference by *C. burnetii* for amino acids over carbohydrates as carbon and energy sources [4]. *C. burnetii* has a biochemically unusual peptidoglycan with associated protease-resistant proteins that may provide protection against CCV degradative activities that can quickly destroy *E. coli* [6], [7].

The cellular microbiology and known metabolic properties of other obligates provide insight into conditions that might support axenic growth. *C. trachomatis* replicates in a vacuole disconnected from the endocytic pathway [8]. The compartment is freely permeable to cytoplasmic ions and has a pH of 7.2 [9]. Vesicular-meditated nutrient delivery is invoked based on vacuole interactions with multivesicular bodies, lipid droplets, and Golgi-derived vesicles [10]. Defined metabolic activities of purified chlamydia include transport and oxidation of glucose-6-phosphate [11]. Vacuoles harboring *E. chaffeensis* and *A. phagocytophilum* resemble early endosomes and autophagosomes, respectively, with predicted pHs slightly lower than neutrality [12], [13]. Intracellular trafficking studies suggest access to ample supplies of amino acids [12], [13]. *Rickettsia* spp. replicate in the well-defined milieu of the host cytoplasm and, similar to *C. trachomatis*, scavenge ATP from the host via the activity of an ATP/ADP translocase [14].

## *In Silico* Pathway Reconstruction Reveals Metabolic Capacity

Along with known metabolic capabilities and host cell niches, clues to axenic growth requirements can be gleaned from *in silico* metabolic pathway reconstructions. An excellent recent review by Fuchs and co-authors [15] details predicted metabolic capacities of *Coxiella*, *Chlamydia*, and *Rickettsia* based on genome data. A common nutritional deficiency of these and other obligates is extensive amino acid auxotrophy that is compensated for by the activities of amino acid and peptide permeases that scavenge amino acids from the host [15]. For example, *C. burnetii* encodes 13 predicted major facilitator superfamily transporters having documented roles in amino acid uptake [16], [17] and several peptide transporters [18]. *C. burnetii* encodes the largest number of open reading frames (2,280 in the Nine Mile reference strain) among the obligates discussed in this review, and consequently, has predicated metabolic complexity relative to these bacteria [18]. Obligates that have undergone more extensive genome reduction rely on additional specialized transport systems to acquire nutrients from the host. For example, genome data indicate defects in purine and pyrimidine biosynthesis by *C. trachomatis*, *R. rickettsia*, *R. prowazekii*, and *O. tsutsugamushi*. Consequently, these organisms have evolved predicted and verified transporters that import host nucleotides [10], [14], [19], [20]. Consistent with defined utilization of glucose-6-phosphate and a largely intact glycolytic pathway, *C. trachomatis* has a predicted transporter (UhpC) for this energized sugar that likely represents an important carbon and energy source [15].

## Do Not Forget the Oxygen

Low oxygen concentration (1–5%) was essential for axenic growth of *C. burnetii*, a result that seems counterintuitive considering the bacterium prodigiously grows in host cells cultivated in ambient oxygen (∼21% O_2_). However, the intracellular oxygen concentration of cultured cells is generally lower than the extracellular concentration [21], and tissues have a range of oxygenation levels that can be well below ambient levels [22].

The impetus for testing low oxygen arose from genome analysis showing *C. burnetii* encodes the terminal oxidases cytochrome *bd* and cytochrome *o.* Thus, *C. burnetii* appeared adaptable to growth under different oxygen concentrations because cytochrome *bd* and cytochrome *o*, based on O_2_ affinities, are typically used under microaerobic and aerobic conditions, respectively. *C. trachomatis*, *R. rickettsia*, and *R. prowazekii* also encode cytochrome *bd*, implying a microaerobic environment might be optimal for axenic growth of these organisms. Other obligates might simply prefer a low oxygen environment to lessen oxidative stress.

## Getting Started

Known pathogen physiology, niche characteristics, and predicted metabolic capacity provide a basis on which to embark on a stepwise approach to axenic media development. Two important technical considerations before beginning are 1) obtaining adequate amounts of highly pure bacteria for testing, and 2) developing a straightforward assay to gauge metabolic fitness. Obligate intracellular bacteria are typically cultivated in tissue culture—a growth system that requires an extensive purification protocol to rid bacterial preparations of contaminating host cell material—with the most problematic contaminates for metabolic studies being mitochondria. Incorporation of radioactive amino acids into protein reflects a biosynthetic process reliant on the activity of major metabolic pathways, and thus is an informative and easy assay of global metabolic activity. *C. burnetii* protein synthesis was measured by scintillation counting and/or gel electrophoresis and autoradiography following incubation in different media formulations containing [^35^S] cysteine-methionine.

The first media component to identify is a metabolically permissive buffer having a pKa near the predicted pH of the bacterium's intracellular niche. Testing of buffers containing [^35^S] cysteine-methionine and readily metabolized glutamate showed that citrate buffer was optimal for *C. burnetii* protein synthesis [23]. Various salt mixtures providing physiologic concentrations of ions can then be tested, again with composition based on intracellular habitats. As speculated, *C. burnetii* preferred serum levels of Na^+^, K^+^, and Cl^−^, and was particularly sensitive to Cl^−^ concentration [23]. The resulting buffer was supplemented with nutrients (e.g., fetal bovine serum [FBS]) predicted to be transported from the host extracellular environment to the CCV via fluid phase endocytosis. Neopeptone was added as the bulk carbon and energy source based on *Coxiella*'s known and predicted preference for amino acids/peptides [23].

During *C. burnetii* media development, metabolic activity continued to improve, but increases in genome equivalents by quantitative PCR were not detected. Thus, to gain insight into potential media deficiencies, the transcriptomes of *C. burnetii* incubated in media and growing in Vero host cells were compared [24]. As expected, the corresponding gene transcriptional profiles were highly discordant. However, a marked down regulation of ribosomal gene expression by axenically cultured bacteria was observed, suggesting that, despite the presence of a rich amino acid source (neopeptone), media was still deficient in amino acids. A different source of amino acids, casamino acids, was then tested. Additionally, media was supplemented with a high concentration (1.5 mM) of L-cysteine based on the similar requirement for axenic growth of *Legionella pneumophila*, a close relative of *C. burnetii*'s. Casamino acids and L-cysteine had an additive effect on metabolic fitness under ambient oxygen (∼21%), but again, bacterial replication was not observed.

**Figure 1:**
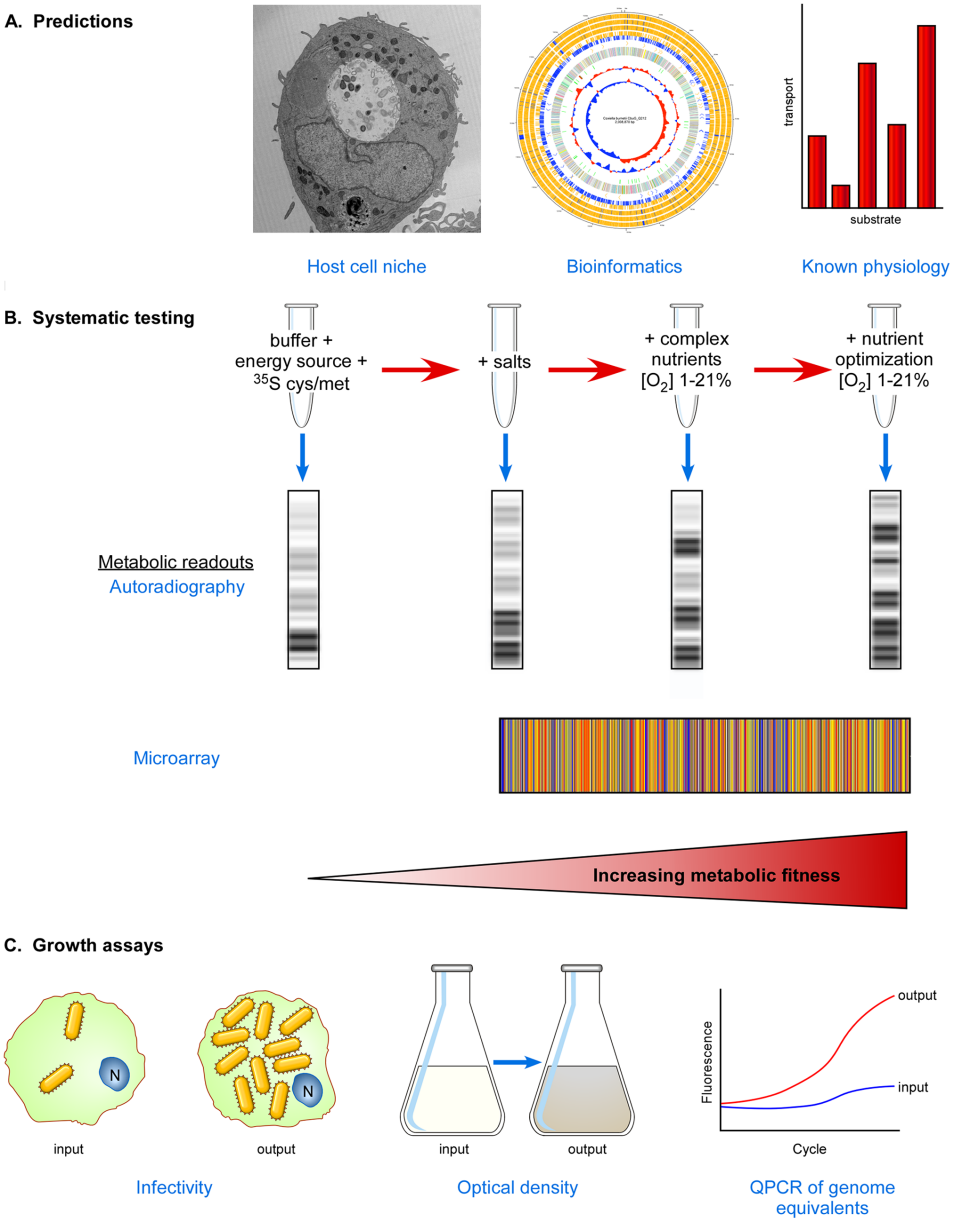
Systematic approach to developing media supporting host cell–free growth of obligate intracellular bacteria. (A) Predictions based on niche characteristics, metabolic pathway reconstructions, and known physiology of purified organisms can be used to establish initial media pH and compositions. Oxygen tension should be tested empirically. (B) Stepwise testing of media formulations and oxygen tension to find conditions that support increased metabolic fitness using informative indicators of metabolic activity such as SDS-PAGE/autoradiography and transcriptional microarrays. Titrations of all media constituents should be conducted since high concentrations of some can be inhibitory [23]. (C) Growth assays to determine if increasing metabolic fitness correlates with increasing bacterial numbers. The infectivity, optical density, and/or genome equivalents of input bacteria can be compared to output bacteria following incubation.

Negative growth results prompted an assessment *C. burnetii* replication at low oxygen levels. When *C. burnetii* was incubated in a medium now termed acidified citrate cysteine medium (ACCM) in 2.5% oxygen, vigorous growth (∼3 log_10_ in 6 days) occurred. A summary of a systematic approach to developing axenic growth media for obligate intracellular bacteria is depicted in Figure 1.

## *Chlamydia*: A Work in Progress

Two recent reports support the idea that axenic growth of *C. trachomatis* may be possible by refuting dogma that the non-replicating, infectious elementary body (EB) is incapable of metabolism outside of a eukaryotic host cell [11], [25]. Haider et al. [25] showed by Raman microspectroscopy and autoradiography that labeled phenylalanine is incorporated by EBs during extended incubation in DGM-21A, a medium that, interestingly, is optimized for growth of *Acanthameoba* sp. Omsland and co-workers [11] subsequently developed a novel phosphate buffer–based CIP-1 medium that supports pronounced metabolism of host cell–free *C. trachomatis*. Based on previous characterization of the chlamydia-containing vacuole [9], CIP-1 has ion concentrations and a pH mimicking the host cytoplasm. Moreover, bioinformatics data and known physiology prompted addition of glucose-6-phosphate and dithiothreitol, as well as FBS, all amino acids, and four nucleotide triphosphates to account for auxotrophies. Seminal findings of this study include (1) glucose-6-phosphate is a preferred energy source of EBs; (2) replicative reticulate bodies (RB), but not EBs, require exogenous ATP as an energy source; and (3) microaerobic conditions enhance metabolic activity.

CIP-1 medium with further modifications might support axenic replication of *C. trachomatis*. However, a potential obstacle is reproducing conditions that promote completion of the chlamydial biphasic developmental cycle. Because only EBs are infectious, failure to accomplish the EB-to-RB-to-EB cycle under axenic conditions would likely result in cultured bacteria that are non-infectious. Differentiation of *L. pneumophila* between replicative and transmissive forms is dependent on stationary phase physiology associated with nutrient limitation [26]. A similar scenario is proposed for developmental transitions of *C. burnetii* wherein non-replicating and metabolically dormant small cell variants (SCVs) differentiate in replicative large cell variants (LCVs) that, in turn, convert back to SCVs [27]. It is logical to suspect chlamydial development is also regulated by nutrient availability and that nutrient-derived developmental signals could be reproduced in axenic media. Indeed, developmental transitions of *C. burnetii* in ACCM appear comparable to those of host cell–grown bacteria [24]. By associating *C. trachomatis* metabolic activities and developmental transitions with modifications of CIP-1 medium, critical insight into medium constituents that impact development will be gained that may ultimately lead to axenic replication that mimics growth in host cells.

## Extension to Unculturable Normal Flora

Although this review emphasizes approaches to culturing obligate intracellular bacterial pathogens, similar logic and strategies can be applied to growth of “unculturable” human normal flora that are refractory to cultivation using conventional techniques [28]. Metagenomic sequencing and other molecular techniques have verified that only a small sub-fraction of the human microbiome has surrendered to conventional culture techniques. As stressed for obligates, replicating the microbe's natural environment as closely as possible can be instrumental to successful culture. This can mean using the environment itself as a culture medium [28]. Sizova and co-workers [29] adapted methods originally designed for culturing of environmental bacteria to culture recalcitrant human oral bacteria. One approach used an ingeniously designed incubation device consisting of a removable oral appliance with agarose-containing diffusion minichambers supporting in vivo growth of a mixed bacterial culture. Subsequent subculture of chamber bacteria on basic anaerobic medium (BM) yielded previously uncultivated taxa. A second successful approach used long-term incubation of microtiter plates where individual wells containing BM were inoculated with single bacterial cells derived from subgingival plaque. The first approach illustrates the poorly understood phenomenon that initial in vivo growth increases the likelihood of subsequent in vitro growth, while the second approach demonstrates the principal that successful culture of previously uncultivated bacteria is enhanced if single cells are allowed to replicate without competition from faster-growing neighbor bacteria [29].

## Concluding Remarks

It is reasonable to reclassify *C. burnetii* as a facultative intracellular bacterium, although this designation can be debated based on the absence of a defined natural environment that sustains extracellular growth [30]. Axenic growth has fueled important new areas of research, including development of a complete set of genetic tools [31]. There is no obvious reason why similar axenic growth cannot be achieved for *Anaplasma*, *Ehrlichia*, *Chlamydia, Orientia*, and *Rickettsia*. With the exception of *Orientia*, these bacteria contain a substantially reduced genome relative to the ∼2 megabase genome of *C. burnetii* that may present a greater barrier to overcome in pursuit of axenic growth. However, a similar systematic approach that exploits known and predicted physiologic behaviors, and persistence in testing, could prove successful in rescuing these obligates from their host cell.
